# Resolution of Lipopolysaccharide-Induced Inflammation Followed by DNA Hypomethylation and Increased Tetrahydrobiopterin Biosynthesis in Mouse Hippocampus

**DOI:** 10.3390/brainsci15080880

**Published:** 2025-08-18

**Authors:** Jennyffer Souza, Debora da Luz Scheffer, Alexandre Francisco Solano, Samantha Veloso, Luisa Cruz, Rodrigo Foganholi-Silva, Alexandra Latini

**Affiliations:** 1 Laboratory of Bioenergetics and Oxidative Stress (LABOX), Department of Biochemistry, Federal University of Santa Catarina, Florianópolis 88037-100, Brazil; 2 Epigenetic Study Center and Gene Regulation, Program in Environmental and Experimental Pathology, Paulista University, São Paulo 01310-100, Brazil

**Keywords:** neopterin, epigenetics, DNA methylation

## Abstract

**Background**: Robust evidence supports the role of tetrahydrobiopterin (BH4) metabolism in sustaining inflammation; however, the mechanisms underlying the persistent upregulation of the BH4 pathway remain incompletely understood. This study investigated the epigenetic regulation of BH4 metabolism following a single injection of lipopolysaccharide (LPS) in the mouse hippocampus. **Methods**: Male C57BL/6J mice received either saline or LPS (0.33 mg/kg, i.p.) and were sacrificed at 4 h or 24 h post injection. Behavioral assessments and analyses of hippocampal neurotransmitter metabolism, DNA methylation profile, oxidative stress, and inflammasome activation were performed. Neopterin levels, a marker of immune system activation, were measured in both the plasma and hippocampus. **Results**: LPS-treated mice exhibited sickness behavior, including reduced locomotor and exploratory activity at both 4 and 24 h. While exploratory behavior showed partial recovery by 24 h, locomotor activity remained impaired. Neopterin levels increased in both the plasma and hippocampus following LPS administration but returned to baseline in the hippocampus by 24 h. Despite the normalization of neopterin, a persistent pro-inflammatory state in the hippocampus was evident at 24 h, as shown by increased expression of Ikbkb and components of the NLRP3 inflammasome, along with elevated oxidative stress markers. Upregulation of *Nrf-2* and *Hmox1* suggested activation of a protective antioxidant response. Dopaminergic metabolism was disrupted, indicating impaired BH4-dependent dopamine turnover. Epigenetic analysis revealed increased expression of DNA methyltransferases (*Dnmt1*, *Dnmt3a*, *Dnmt3b*) and *Tet2*, along with reduced expression of *Tet1* and *Tet3*. Promoter hypomethylation of *Gch1* and *Ptps* was observed, correlating with increased hippocampal expression and potentially elevated BH4 levels. **Conclusions**: Together, these findings show that a single LPS challenge was sufficient to induce the activation of the BH4 synthesis pathway during the late acute inflammatory phase, both systemically and in the hippocampus, potentially driven by epigenetic modifications such as promoter hypomethylation. This may contribute to the perpetuation of neuroinflammation.

## 1. Introduction

Tetrahydrobiopterin (BH4) is an essential cofactor for the enzymes phenylalanine hydroxylase, tyrosine hydroxylase, tryptophan hydroxylase, alkylglycerol monooxygenase, and all forms of nitric oxide synthase (NOS). Consequently, BH4 plays a critical biochemical role in nitric oxide synthesis, phenylalanine catabolism, ether lipid metabolism, and the production of the neurotransmitters dopamine and serotonin [for a review see [1]]. Intracellular BH4 levels are finely regulated by three biosynthetic pathways: the de novo, salvage, and recycling pathways. The de novo pathway is initiated by GTP cyclohydrolase I (GTPCH), the first rate-limiting enzyme, whose activity is largely determined by the expression of *GCH1*. The expression of this gene is strongly induced under inflammatory conditions, such as exposure to interferon-γ (IFN-γ), lipopolysaccharides (LPSs), or interleukin-1 (IL-1β) [2]. However, because downstream enzymes in the pathway are not similarly upregulated by inflammation, a metabolic bottleneck occurs, resulting in the accumulation of both neopterin and BH4 [3]. Elevated plasma neopterin levels are widely recognized as a sensitive biomarker of cell-mediated immune activation and have long been used to monitor immune system activity [4,5]. The final steps in the de novo pathway involve the enzymes 6-pyruvoyl tetrahydropterin synthase (PTPS) and sepiapterin reductase (SPR). The salvage pathway converts sepiapterin, generated from precursors of the de novo pathway, into BH4 through the action of SPR and dihydrofolate reductase (DHFR) [1,6]. In tissues with a high BH4 demand, such as the liver, the recycling pathway is critical. This pathway regenerates BH4 from its oxidized form (qBH2), which accumulates after BH4 functions as a cofactor. The last step of this conversion is catalyzed catalysis by dihydropteridine reductase (DHPR).

Given its essential roles in multiple physiological processes, the regulation of BH4 metabolism is of critical importance. BH4 is required for nitric oxide (NO) synthesis, a key mediator of vascular relaxation [7], and for phenylalanine hydroxylation, which prevents the toxic accumulation of phenylalanine by facilitating its conversion to tyrosine [8]. Additionally, BH4 is indispensable for the enzymatic activity of tyrosine hydroxylase and tryptophan 5-hydroxylase, enzymes responsible for the synthesis of the neurotransmitters dopamine and serotonin [9,10]. More recently, we have shown that BH4 is also required for immune cell expansion and tissue infiltration in preclinical systems characterized by chronic inflammation [11]. Dysregulation of BH4 metabolism can therefore have wide-ranging pathological consequences. Understanding the molecular mechanisms that control BH4 availability, particularly under inflammatory conditions, is crucial for elucidating its role in health and disease.

Gene expression is tightly regulated by epigenetic mechanisms, including DNA methylation, histone modifications, and noncoding RNA-mediated processes, all of which contribute to the establishment of cell type-specific transcriptional programs [12]. Among these, DNA methylation is one of the most extensively studied modifications. It involves the covalent addition of a methyl group to the fifth carbon of cytosine residues within CpG dinucleotides, forming 5-methylcytosine (5mC). This reaction is catalyzed by DNA methyltransferases, including DNMT1, which maintains existing methylation patterns, and DNMT3A and DNMT3B, which are responsible for de novo methylation [13]. Conversely, active DNA demethylation is mediated by the ten-eleven translocation (TET) family of enzymes, TET1, TET2, and TET3, which oxidize 5mC to 5-hydroxymethylcytosine (5hmC), initiating a process that ultimately restores unmethylated cytosines [14]. The dynamic balance between methylation and demethylation is essential for proper gene expression regulation, and disruptions to this equilibrium are increasingly recognized as contributors to disease pathogenesis.

Increasing evidence indicates that aberrant DNA methylation contributes to the onset and progression of various neurodegenerative diseases. For example, elevated DNA methylation has been implicated in motor neuron apoptosis in amyotrophic lateral sclerosis (ALS) [15], as well as in neuronal dysfunction and death via downregulation of PGC-1α in patients with Parkinson’s disease [16]. In the human brain, DNA methylation is essential for normal development and function, playing critical roles in synaptic plasticity, neuronal repair, learning, and memory [17]. Conversely, dysregulated DNA demethylation is also associated with neuropathology. In Parkinson’s disease, hypomethylation of the tumor necrosis factor alpha (*TNF-α)* promoter in the substantia nigra has been linked to increased inflammatory signaling and disease progression [18]. Similarly, individuals affected by Alzheimer’s disease exhibit hypomethylation of pro-inflammatory genes such as *TNFα* [19] and *Il6* [20], potentially contributing to neuronal damage and chronic neuroinflammation. Together, these findings underscore the importance of balanced DNA methylation dynamics in maintaining neuronal integrity and highlight their relevance in the pathogenesis of neurodegenerative disorders.

Given the growing evidence supporting that DNA methylation plays a critical role in the pathophysiology of diseases characterized by chronic inflammation, we hypothesized that the BH4 synthesis pathway may be subject to epigenetic regulation via changes in DNA methylation. Specifically, we propose that DNA methylation dynamics could modulate the expression of BH4-related genes, thereby contributing to the sustained upregulation of this pathway following an acute inflammatory challenge. Such a mechanism may help explain how a single pro-inflammatory insult can lead to a prolonged inflammatory response through persistent metabolic reprogramming.

## 2. Material and Methods

### 2.1. Animals

Adult Swiss mice (3–5 months old; body mass 45–50 g) from the central animal facility of the Biological Sciences Center at the Federal University of Santa Catarina (UFSC) were kept in a controlled environment (22 ± 1 °C, 12 h light/dark cycle) with free access to water and food. The experimental protocols were approved by the Animal Research Ethics Committee of UFSC (CEUA 9469201118; approval date: 4 June 2019). To minimize variability associated with hormonal cycles, we used only male mice in this study.

### 2.2. LPS-Induced Inflammatory Process

Acclimated mice were randomly divided into four groups (n = 5–10 animals; the exact number of animals per group for each experimental condition is presented in Table S1). The vehicle 4 h group and the vehicle 24 h group received an intraperitoneal (i.p.) injection of 0.9% sodium chloride (NaCl) (0.1 mL per 10 g of body weight) and were sacrificed after 4 h or 24 h post administration. The LPS 4 h group and the LPS 24 h group received an i.p. injection of LPS (0.33 mg/kg body weight; E. coli LPS, serotype 0127) and were euthanized after 4 h or 24 h post administration. Euthanasia was performed by cervical dislocation, followed by the collection of plasma and hippocampus. The hippocampus was specifically analyzed because inflammatory conditions triggered by LPS are extensively described as triggers of neuroinflammation, a condition that affects memory formation and learning, processes for which the hippocampus is a key brain region. The LPS dosage was based on previously published data [21,22].

### 2.3. Locomotor Activity

Spontaneous locomotor activity was assessed using the open field test. After familiarization session with the apparatus (white round with 40 cm diameter and 30 cm height), the animals were placed in the center of the open field apparatus and allowed to explore the arena for 5 min. Locomotor activity and rearing frequencies were recorded with a digital camera, and the results were evaluated using ANYmaze^®^ software (https://www.any-maze.com/contact/#usa-international, accessed on 28 June 2025, Stoelting, Wood Dale, IL, USA). After each animal’s test, the arena was thoroughly cleaned with 20% ethanol solution and dried with paper towels before introducing the next animal. The experiment was conducted under low light conditions (12 lux) as previously reported [23]. The animals were evaluated both before (basal) and 4 or 24 h after the administration of LPS/vehicle.

### 2.4. Determination of Neopterin Levels by High-Performance Liquid Chromatography (HPLC)

Plasma proteins were precipitated by adding an equal volume of 5% trichloroacetic acid (TCA). Hippocampal samples were first homogenized (1:5, *w*/*v*) in 60 mM potassium phosphate buffer and centrifuged at 3000× *g* for 10 min at 4 °C. The resulting supernatants were used for protein quantification. To precipitate proteins, an equal volume of 5% TCA was added to the supernatants, followed by centrifugation at 15,000× *g* for 10 min at 4 °C. Subsequently, 20 µL of the clarified supernatant was filtered through a 0.22 µm membrane filter and transferred to vials for analysis by HPLC (Alliance e2695, Waters, Milford, CT, USA). Neopterin levels were analyzed by HPLC using a Supelcosil™ LC-18-T reverse-phase column (15 × 4.6 mm, 5 µm particle size). The mobile phase consisted of 85% 15 mM potassium phosphate buffer and 15% acetonitrile (pH 6.4), with isocratic elution at a flow rate of 0.7 mL/min. The column temperature was maintained at 35 °C. Neopterin was identified and quantified using a multi-wavelength fluorescence detector (2475 module, Waters, Milford, CT, USA) set at an excitation wavelength of 355 nm and an emission wavelength of 438 nm, as previously described [24]. The results were expressed as nmol/L for plasma samples and as nmol/mg of protein for hippocampal tissue.

### 2.5. Determination of Monoamine Levels by HPLC

The monoamines dopamine and serotonin, along with their respective metabolites, 3,4-dihydroxyphenylacetic acid (DOPAC) and 5-hydroxyindoleacetic acid (5-HIAA), were analyzed in the hippocampus using HPLC (Alliance e2695, Waters, Milford, CT, USA) coupled with electrochemical detection (Waters 2465, Waters, Milford, CT, USA) set at +400 mV, as previously described [25]. Hippocampal tissue was homogenized (1:5, *w*/*v*) in 60 mM TFK buffer. Hippocampal samples were first homogenized (1:5, *w*/*v*) in 60 mM potassium phosphate buffer and centrifuged at 3000× *g* for 10 min at 4 °C. The resulting supernatants were used for protein quantification. To precipitate proteins and preserve the stability of the metabolites, the supernatants were mixed (1:1, *v*/*v*) with 0.1 M perchloric acid containing 0.02% sodium metabisulfite and centrifuged at 16,000× *g* for 10 min at 4 °C. Subsequently, 100 µL of the clarified supernatant was filtered through a 0.22 µm membrane filter and transferred to vials for analysis by HPLC. Chromatographic separation was carried out using a C18 reverse-phase column (Synergi Hydro, 150 × 2.0 mm, 4 µm; Phenomenex, Torrance, CA, USA) maintained at 30 °C. A 20 µL aliquot of each sample was injected, and analytes were eluted using an isocratic mobile phase composed of 90 mM sodium phosphate, 50 mM citric acid, 1.7 mM sodium 1-heptanesulfonate, 50 µM EDTA, and 10% acetonitrile (pH 3.0) at a flow rate of 0.25 mL/min. Dopamine, DOPAC, serotonin, and 5-HIAA levels were quantified and expressed as ng/mg of protein.

### 2.6. Measurement of Reactive Oxygen Species (ROS)

ROS were assessed using nitroblue tetrazolium (NBT), which reacts predominantly with superoxide anion to form formazan, a blue-colored compound [26]. Briefly, samples were incubated with 0.5% NBT at 37 °C for 45–60 min. Positive control samples were prepared by adding 150 µL of 1.5 mM NADPH. Following incubation, the samples were centrifuged at 900× *g* for 10 min. The resulting pellet was washed with 500 µL of phosphate-buffered saline. To solubilize the formazan product, 200 µL of a potassium hydroxide plus dimethylsulfoxide (DMSO) solution (prepared by mixing 1800 µL of 2 M KOH with 2100 µL of DMSO) was added. Then, 100 µL of the solubilized solution was transferred to a 96-well plate, and absorbance was measured at 630 nm. A standard curve prepared with known concentrations of formazan was used for quantification. The results were expressed as µg of formazan/mg of protein.

### 2.7. Measurement of Thiobarbituric Acid-Reactive Substances (TBA-RSs)

TBA-RS levels in the mouse hippocampus were determined as previously described by our group [27]. Hippocampal samples were homogenized in acetic acid buffer containing 0.45% sodium dodecyl sulfate (SDS) and centrifuged at 10,000× *g* for 10 min. In glass tubes, 120 µL of the supernatant was mixed with 120 µL of 0.67% thiobarbituric acid (TBA) and thoroughly homogenized. Samples were incubated in a water bath at 95 °C for 60 min, then cooled in an ice bath for 5 min. After cooling, 400 µL of butanol was added, and the mixture was vortexed for 20 s. Samples were then centrifuged at 5000× *g* for 5 min. A 200 µL aliquot of the upper organic phase was transferred to a 96-well plate for spectrophotometric analysis at 532 nm. Quantification was performed using a standard curve prepared with known concentrations of 1,1,3,3-tetramethoxypropane. TBA-RS levels were expressed as nmol/mg of protein.

### 2.8. Determination of NO Levels

Nitric oxide (NO) levels were assessed indirectly by quantifying its stable metabolite, nitrite, using the Griess reaction [28]. Hippocampal tissues were homogenized in phosphate-buffered saline containing 1 mM EDTA (1 mL per 100 mg of tissue). Briefly, 25 µL of the hippocampal homogenate was mixed with 25 µL of 1% sulfanilamide in 5% phosphoric acid and incubated at room temperature for 10 min in the dark. Then, 25 µL of 1% N-(1-naphthyl)ethylenediamine dihydrochloride solution was added, followed by an additional 10-min incubation period in the dark. Absorbance was measured at 540 nm using a microplate reader. Nitrite concentrations were determined based on a standard curve prepared with known concentrations of sodium nitrite and expressed as µmol/mg of protein.

### 2.9. Total RNA Extraction

Total RNA was isolated from the hippocampus using the TRIzol^®^/chloroform/isopropanol method. Tissue samples were homogenized in 0.5 mL of TRIzol^®^ reagent (15596026, Invitrogen, Carlsbad, CA, USA), and phase separation was performed by adding 0.2 mL of chloroform (102444, Merck, Rahway, NJ, USA), followed by centrifugation at 17,000× *g* for 15 min at 4 °C. The aqueous phase was carefully collected into new tubes, and the interphase and organic phase were discarded. RNA was precipitated by adding 0.5 mL of cold absolute isopropanol (109634, Merck, Rahway, NJ, USA), followed by incubation at room temperature for 10 min and centrifugation at 17,000× *g* for 15 min. The resulting RNA pellet was washed with 75% ethanol, and the supernatant was removed via gentle inversion. The pellet was air-dried and resuspended in 20 µL of DEPC-treated water. RNA concentration and purity were assessed spectrophotometrically at 260 and 280 nm using a NanoDrop 2000 (Thermo Scientific, Uniscience, Waltham, MA, USA). The RNA samples were stored at −80 °C until further use.

### 2.10. cDNA Synthesis

cDNA was synthesized from 2000 ng of total RNA using reverse transcription with 2 U of reverse transcriptase (SuperScript™ II Kit, 18064022, Invitrogen, Carlsbad, CA, USA), following the manufacturer’s instructions. First-strand synthesis was performed in a final reaction volume of 20 µL containing 500 µM dNTPs, 25 µg/mL oligo(dT) primers, 1× first-strand buffer, 40 U of RNase inhibitor, 10 µM DTT, and the SuperScript™ II enzyme. To denature secondary RNA structures, the RNA, water, and primer mixture were first incubated at 65 °C for 5 min. The reverse transcription reaction was then carried out at 50 °C for 50 min, followed by enzyme inactivation at 85 °C for 5 min. The resulting cDNA was diluted to a final concentration of 100 ng/µL and stored at −20 °C until use.

### 2.11. Gene Expression by Quantitative Real-Time PCR (qPCR)

The primer sequences (Exxtend, Campinas, SP, Brazil) and PCR conditions are listed in Table 1. Each qPCR reaction was performed in a final volume of 10 µL, containing 5 µL of SYBR Green I Master Mix, 0.4 µM of each specific primer, 1 µL of synthesized cDNA, and RNase-free Milli-Q water. Relative gene expression levels were calculated using the comparative threshold cycle (Ct) method (2^−ΔCt^). For this, the average Ct value of each target gene was subtracted from the average Ct of the reference genes, generating ΔCt values. Expression levels were then calculated using the 2^−ΔCt^ formula, and the results were expressed as the ratio of target gene expression to the geometric mean of the reference genes *Gapdh* and *β-actin*. Comparisons between experimental and control groups were made using the ΔΔCt method.

### 2.12. Genomic DNA Extraction

Genomic DNA was extracted from the hippocampus using a phenol/chloroform/isoamyl alcohol method, as previously described by our group [29]. Briefly, hippocampal tissue was homogenized in extraction buffer (10 mM Tris, pH 3.0; 0.5% SDS; 5 mM EDTA) and incubated with 20 mg/mL proteinase K at 56 °C for 16 h. DNA was isolated by adding 500 µL of equilibrated phenol to the lysate, followed by centrifugation at 1700× *g* for 15 min. The upper aqueous phase was transferred to a new tube and mixed with 200 µL of chloroform. After centrifugation under the same conditions, the resulting supernatant was collected, and DNA was precipitated by adding 800 µL of isopropanol and 150 µL of 3 M sodium acetate. The mixture was centrifuged at 1700× *g* for 15 min, and the pellet was washed with 500 µL of 70% ethanol, followed by a final centrifugation at 17,000× *g* for 15 min. After discarding the supernatant, the DNA pellet was air-dried and resuspended in 50 µL of nuclease-free water. DNA concentration and purity were assessed spectrophotometrically at 260 and 280 nm using a NanoDrop 2000 (Thermo Scientific, Uniscience).

### 2.13. DNA Methylation in the Promoter Gene

**Scheme 1:**
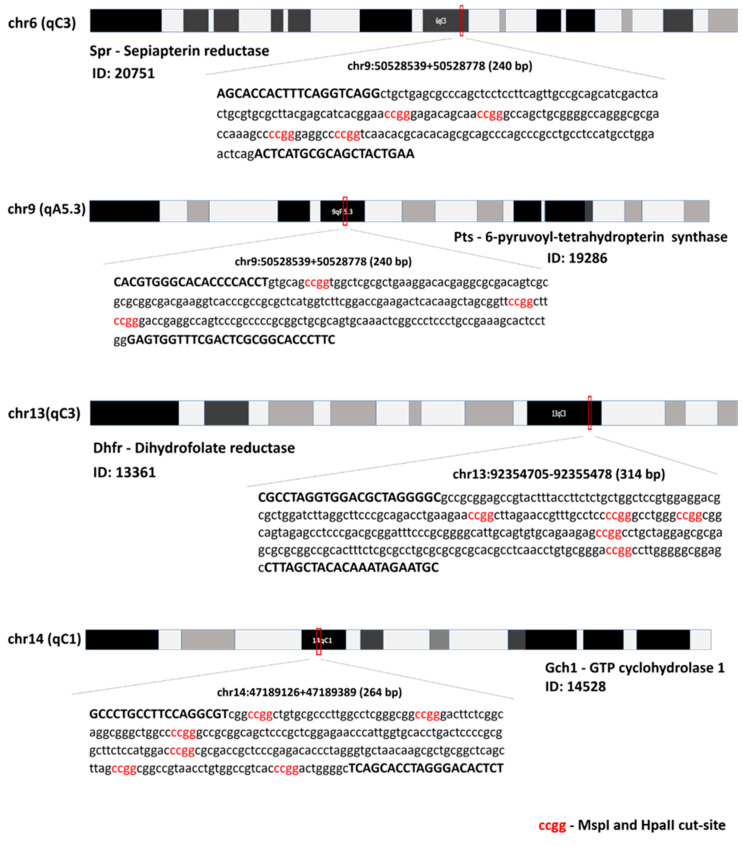
A schematic representation of the amplified promoter regions of genes involved in the tetrahydrobiopterin (BH4) synthesis pathway for DNA methylation analysis. Each panel shows the chromosomal location of the target gene and the specific region of the promoter that was amplified. Primer sequences are indicated in bold uppercase letters, and restriction sites for the endonucleases *MspI* and *HpaII* are marked in red. These sites were used to assess CpG methylation status via differential enzyme sensitivity.

For the analysis of 5-methylcytosine (5-meC) and 5-hydroxymethylcytosine (5-hmeC), the same concentration of gDNA was treated with T4-β-glucosyltransferase (M0357L, New England BioLabs, Ipswich, MA, USA), which adds a glucose unit to 5-hmeC to distinguish between methylation and hydroxymethylation of DNA. The samples were then digested with the restriction enzymes MspI or HpaII (R0106L, New England BioLabs,, Ipswich, MA, USA). Following DNA treatment, qPCR reactions were performed in 40 amplification cycles in a total volume of 10 μL, containing PowerUp™ SYBR™ Green Master Mix 2× (5 μL; A25741, Applied Biosystems, Foster City, CA, USA), 0.5 μM of each primer, 1 μL of treated gDNA, and nuclease-free water. Primers were designed in regulatory regions (DNaseI hypersensitivity cluster sites, marked by histone modifications, CpG regions, and transcription factor binding sites) using Primer3 Input software (version 0.4.0). Primers were verified using in silico PCR (https://genome.ucsc.edu/, accessed on 12 August 2025), and the characteristics of the primers and gene regions analyzed are illustrated in Scheme 1. The levels of 5-meC or 5-hmeC were determined using the cycle threshold (Ct) method, and the methylation results are presented as *HpaII* levels-*MspI* levels/control levels, while the hydroxymethylation results are presented as *MspI* levels/control levels.

To assess the levels of 5meC and 5hmeC, equal concentrations of genomic DNA were treated with T4-β-glucosyltransferase (M0357L, New England BioLabs, Ipswich, MA, USA), which selectively glucosylates 5hmeC, enabling differentiation between DNA methylation and hydroxymethylation, as previously reported by our lab [30]. Treated samples were subsequently digested with the restriction enzymes *MspI* or *HpaII* (R0106L, New England BioLabs, Ipswich, MA, USA). qPCR was performed following DNA digestion using 40 amplification cycles in a final volume of 10 μL, containing 5 μL of PowerUp™ SYBR™ Green Master Mix 2× (A25741, Applied Biosystems, Foster City, CA, USA), 0.5 μM of each primer, 1 μL of treated genomic DNA, and nuclease-free water. Primers were designed to target regulatory regions, including DNase I hypersensitive sites, CpG islands, and regions marked by histone modifications and transcription factor binding sites, using Primer3 Input (version 0.4.0). Primer specificity was verified via in silico PCR analysis (https://genome.ucsc.edu/). The primer sequences and gene targets are detailed in Scheme 1.

Relative levels of 5meC and 5hmeC were calculated using the cycle threshold (Ct) method. Methylation levels are expressed as (*HpaII Ct*–*MspI Ct*)/control levels, while hydroxymethylation levels are expressed as *MspI Ct*/control levels.

### 2.14. Protein Determination

Protein concentration was determined according to the Lowry method using bovine serum albumin as the standard [31].

### 2.15. Statistical Analysis

Data are presented as the mean ± standard error of the mean (SEM). The normality of the data distribution was assessed using the Shapiro–Wilk test prior to the application of ANOVA or Student’s *t* test. One-tailed *t* tests were used for behavioral data, as the hypothesis was directional and specifically predicted a reduction in locomotor activity in LPS-treated animals. All other comparisons were conducted using two-tailed tests. ANOVA followed by Tukey’s post hoc test was used to analyze neopterin levels in the plasma and hippocampus. Statistical significance was set at *p* < 0.05. All statistical analyses and graph generation were performed using GraphPad Prism 9^®^ (GraphPad Software, Boston, MA, USA).

## 3. Results

### 3.1. LPS Administration Induced Sickness Behavior, Hippocampal Inflammation, and Oxidative Stress, Increased BH4 Synthesis, and Disrupted Dopamine Turnover

**Figure 1:**
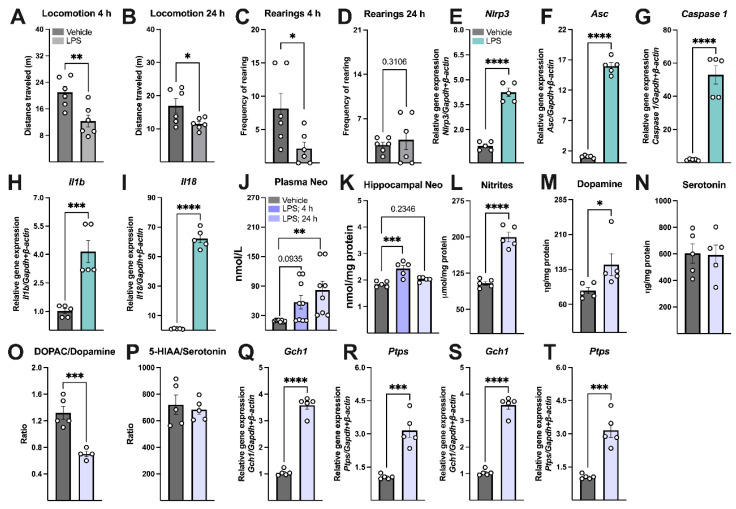
Lipopolysaccharide (LPS) administration induced sickness behavior, hippocampal inflammation, and oxidative stress, increased tetrahydrobiopterin (BH4) synthesis, and disrupted dopamine turnover. Adult male C57BL/6J mice received a single intraperitoneal dose of LPS (0.33 mg/kg), and measurements were performed at 4 h and/or 24 h post injection. Control animals received an equivalent volume of saline solution as vehicle. Total travelled distance (**A**,**B**) and rearing frequency (**C**,**D**) at 4 h and 24 h after LPS administration. NLRP3 inflammasome activation was assessed by measuring the expression of *Nlrp3* (**E**), *Asc* (**F**), *Caspase 1* (**G**), *Il1b* (**H**), and *Il18* (**I**) in the hippocampal tissue at 24 h after LPS administration. Plasma (**J**) and hippocampal levels (**K**) of neopterin at 4 h and 24 h after LPS administration. The levels of nitrites (**L**), dopamine (**M**), and serotonin (**N**) were measured 24 h after LPS administration as indicators of BH4-dependent metabolic activation. Turnover of dopamine (**O**) and serotonin (**P**) was assessed 24 h after LPS administration by measuring the levels of their respective metabolites. Upregulation of BH4 metabolism was assessed by measuring the expression of *Gch1* (**Q**), *Ptps* (**R**), *Spr* (**S**), and *Dhfr* (**T**). Bars represent the mean ± SEM (n = 5–10 animals per experimental group). * *p* < 0.05, ** *p* < 0.01, *** *p* < 0.001, and **** *p* < 0.0001: vehicle vs. LPS. Student’s *t* test for independent samples. One-way ANOVA followed by the Dunnett post hoc test for (**J**,**K**).

Figure 1 shows the induction of sickness behavior, hippocampal inflammation, and oxidative stress, and impaired dopamine turnover following LPS administration. Figure 1A,B show that LPS exposure significantly impaired spontaneous locomotion at both 4 h and 24 h post challenge (4 h = [*t*_(10)_ = 3.32, *p* < 0.01; *d* = 0.52]; 24 h = [*t*_(10)_ = 2.36, *p* < 0.05; *d* = 0.36]). Additionally, vertical exploration was compromised, as indicated by a reduced rearing frequency at 4 h after LPS administration, with no differences observed at 24 h (4 h = [*t*_(10)_ = 2.42, *p* < 0.05; *d* = 0.37]; 24 h = [*t*_(10)_ = 0.51, *p* > 0.05; *d* = 0.02]) (Figure 1C,D). The inflammatory response induced by LPS is characterized by the upregulation of the NLRP3 inflammasome components. Figure 1E–G show significantly increased expression of the receptor *Nlrp3* [*t*_(8)_ = 11.72, *p* < 0.0001; *d =* 0.94], the adaptor protein *Asc* [*t*_(8)_ = 25.06, *p* < 0.0001; *d =* 0.99], and the effector enzyme *Caspase 1* [*t*_(8)_ = 9.25, *p* < 0.0001; *d =* 0.91]. Consistently, expression of the cytokines processed by the NLRP3 inflammasome, *Il1β* [*t*_(8)_ = 5.17, *p* < 0.001; *d =* 0.77] and *Il18* [*t*_(8)_ = 21.28, *p* < 0.0001; *d =* 0.98], was also significantly increased (Figure 1H,I). Neopterin levels, a biomarker of immune system activation, were significantly elevated in response to LPS, with increased levels detected at 24 h in the plasma (Figure 1J) [*F*_(2, 22)_ = 5.50, *p* < 0.05; *d* = 0.33], and at both 4 h and 24 h in the hippocampus [*F*_(2,12)_ = 13.11, *p* < 0.001; *d* = 0.69] (Figure 1K). Figure 1L–P show that BH4-dependent metabolic pathways were also modulated in the hippocampus of LPS-treated mice. Nitrite levels, stable metabolite of NO, were significantly increased at 24 h [*t*_(8)_ = 8.99, *p* < 0.0001; *d =* 0.91] (Figure 1L), as were dopamine levels [*t*_(8)_ = 3.80, *p* < 0.01; *d =* 0.64] (Figure 1M). Serotonin levels remained unchanged [*t*_(8)_ = 1.39, *p* > 0.05; *d =* 0.19] (Figure 1N). Dopamine turnover was significantly reduced [*t*_(7)_ = 5.36, *p* < 0.001; *d =* 0.80] (Figure 1O), whereas serotonin turnover remained unaltered [*t*_(8)_ = 0.48, *p* > 0.05; *d =* 0.01] (Figure 1P). Furthermore, LPS exposure significantly upregulated the expression of genes involved in de novo BH4 biosynthesis, including *Gtpch* [*t*_(8)_ = 15.84, *p* < 0.0001; *d =* 0.97] (Figure 1Q), *Ptps* [*t*_(8)_ = 6.27, *p* < 0.001; *d =* 0.83] (Figure 1R), and *Spr* [*t*_(8)_ = 18.32, *p* < 0.0001; *d =* 0.98] (Figure 1S). Increased expression of *Dhfr* [*t*_(8)_ = 6.30, *p* < 0.0001; *d =* 0.83] (Figure 1T) indicates activation of the BH4 salvage pathway.

**Figure 2:**
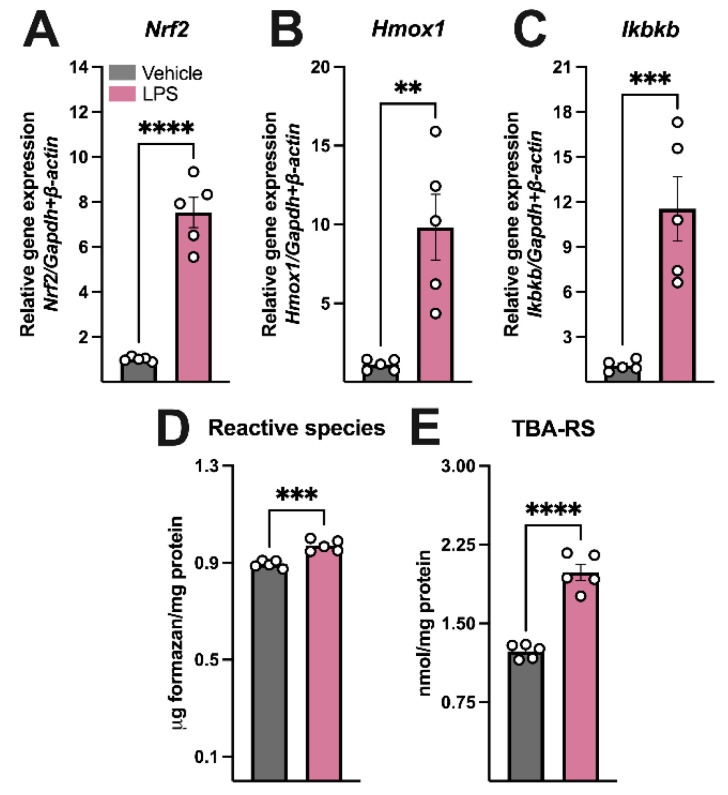
Lipopolysaccharide (LPS) administration induced oxidative stress in the mouse hippocampus. Adult male C57BL/6J mice received a single LPS intraperitoneal dose (0.33 mg/kg) and the measurements were performed after 24 h. Control animals received saline solution as vehicle. Oxidative stress status was assessed by measuring the expression of *Nrf2* (**A**), *Hmox1* (**B**), and *Ikbkb* (**C**) and the levels of reactive species (**D**) and thiobarbituric acid reactive substances (TBA-RSs) (**E**). Bars represent the mean ± SEM (n = 5–10 animals per experimental group). ** *p* < 0.01, *** *p* < 0.001, and **** *p* < 0.0001: vehicle vs. LPS. Student’s *t* test for independent samples.

Figure 2 shows that the pro-inflammatory response triggered by LPS was accompanied by increased oxidative stress. As shown in Figure 2A, the hippocampal expression of *Nrf-2*, the master regulator of the antioxidant and cytoprotective responses, was significantly increased 24 h after LPS administration [*t*_(8)_ = 9.68, *p* < 0.0001; *d =* 0.92]. Similarly, hippocampal expression of *Hmox1* [*t*_(8)_ = 4.18, *p* < 0.01; *d =* 0.69] and *Ikbkb* [*t*_(8)_ = 4.89, *p* < 0.001; *d =* 0.75] was also elevated, as shown in Figure 2B,C, respectively, indicating activation of protective mechanisms against oxidative damage. In line with these findings, Figure 2D,E show increased hippocampal levels of reactive species [*t*_(8)_ = 6.12, *p* < 0.001; *d =* 0.82], and TBA-RS content [*t*_(8)_ = 9.06, *p* < 0.0001; *d =* 0.91], further supporting the presence of oxidative stress.

### 3.2. LPS Modulated the Expression of Key Regulators of DNA Methylation

**Figure 3:**
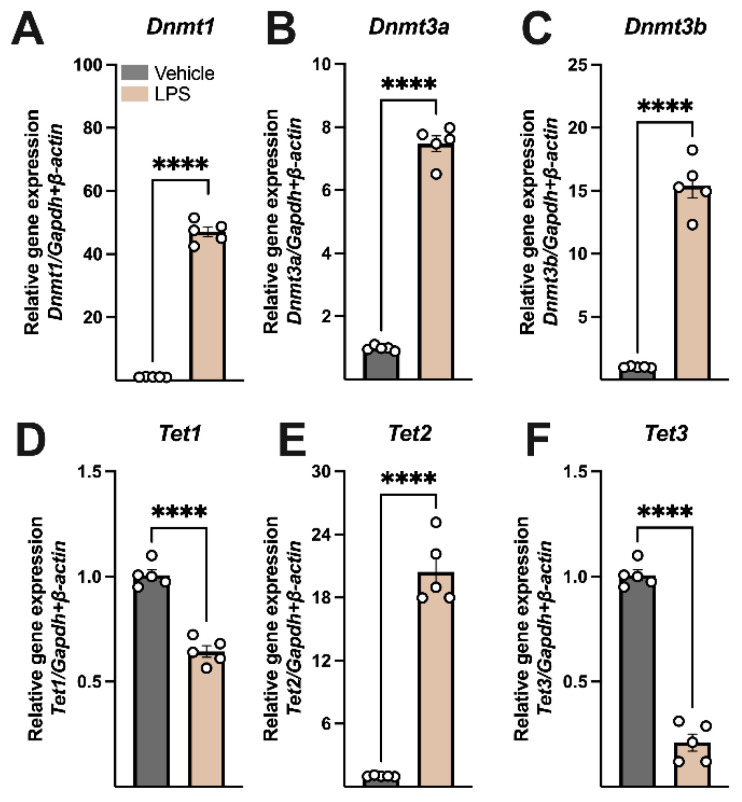
Lipopolysaccharide (LPS) administration modulated the expression of genes involved in DNA methylation. Adult male C57BL/6J mice received a single LPS intraperitoneal dose (0.33 mg/kg) and the measurements were performed after 24 h. Control animals received saline solution as vehicle. DNA methylation was assessed by measuring the expression of genes encoding *Dnmt1* (**A**), *Dnmt3a* (**B**), *Dnmt3b* (**C**), *Tet1* (**D**), *Tet2* (**E**), *and Tet3* (**F**). Bars represent the mean ± SEM (n = 5 animals per experimental group). **** *p* < 0.0001: vehicle vs. LPS. Student’s *t* test for independent samples.

Figure 3 shows that LPS administration altered the expression of enzymes involved in the regulation of DNA methylation. The expression of the genes encoding the maintenance methylation enzyme *Dnmt1* [*t*_(8)_ = 29.74, *p* < 0.0001; *d =* 0.99] (Figure 3A), as well as the de novo methylation enzymes *Dnmt3a* [*t*_(8)_ = 25.34, *p* < 0.0001; *d =* 0.99] (Figure 3B) and *Dnmt3b* [*t*_(8)_ = 15.02, *p* < 0.0001; *d =* 0.97] (Figure 3C), was significantly increased 24 h after the LPS challenge. In contrast, LPS treatment reduced the expression of the DNA demethylation enzymes *Tet1* [*t*_(8)_ = 9.67, *p* < 0.0001; *d =* 0.92] (Figure 3D), and *Tet3* [*t*_(8)_ = 16.57, *p* < 0.0001; *d =* 0.97] (Figure 3F), while increasing the expression of *Tet2* [*t*_(8)_ = 13.84, *p* < 0.0001; *d =* 0.96] (Figure 3E).

LPS administration decreased DNA methylation in the promoter regions of genes associated with BH4 biosynthesis

**Figure 4:**
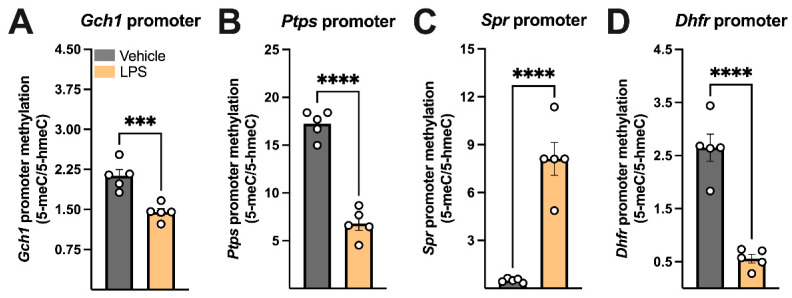
Lipopolysaccharide (LPS) administration modulated DNA methylation in promoter regions of genes involved in the tetrahydrobiopterin (BH4) metabolic pathway. Adult male C57BL/6J mice received a single LPS intraperitoneal dose (0.33 mg/kg) and the measurements were performed after 24 h. Control animals received saline solution as vehicle. The degree of methylation of CpU islands was assessed as a ratio between 5-methyl-cytosine and 5-hydroxy-methyl-cytosine (5-meC/h-hmeC). The methylation of the promoters of *Gch1* (**A**), *Ptps* (**B**), *Spr* (**C**), and *Dhfr* (**D**) was measured. Bars represent the mean ± SEM (n = 5 animals per experimental group). *** *p* < 0.001 and **** *p* < 0.0001: vehicle vs. LPS. Student’s *t* test for independent samples.

Figure 4 shows that LPS administration altered the methylation status of promoter regions of genes encoding BH4 biosynthetic enzymes. Significant hypomethylation was observed in the promoter regions of *Gch1* [*t*_(8)_ = 4.97, *p* < 0.01; *d =* 0.76] (Figure 4A), *Ptps* [*t*_(8)_ = 11.02; *p* < 0.0001; *d =* 0.94] (Figure 4B), *Spr* [*t*_(8)_ = 7.41; *p* < 0.0001; *d =* 0.87] (Figure 4C), and *Dhfr* [*t*_(8)_ = 7.82; *p* < 0.0001; *d =* 0.88] (Figure 4D) 24 h after LPS administration.

## 4. Discussion

This study investigated the potential epigenetic regulation of BH4 synthesis following an acute inflammatory insult induced by LPS. Our findings demonstrate that LPS administration significantly modulated DNA methylation patterns in the promoter regions of genes involved in BH4 biosynthesis in the mouse hippocampus 24 h post challenge. This epigenetic modulation occurred in parallel with a robust pro-inflammatory response, as evidenced clinically by sickness behavior and biochemically by the upregulation of NLRP3 inflammasome components, elevated levels of associated cytokines, increased oxidative stress, and disruptions in dopamine metabolism.

Epigenetic changes are increasingly recognized as key contributors to the development of neurodegenerative diseases and the brain’s vulnerability to acute injury [for a review see [32]]. Modifications to DNA and histones, along with the action of noncoding RNAs, work together to regulate gene expression, enabling the brain to respond and adapt to environmental challenges and injury. These epigenetic mechanisms affect cellular processes such as neurogenesis and DNA repair, as well as more complex functions including brain organization, memory, motor skills, and cognition. Consequently, disruptions in epigenetic regulation can drive the progression of numerous neurological conditions, even in the absence of direct genetic mutations.

Acute inflammation is often characterized by dynamic changes in DNA methylation patterns, particularly leading to hypomethylation of specific gene promoters, which in turn promotes the upregulation of pro-inflammatory interleukins. DNA methylation typically acts as a repressive mark, meaning that a decrease in methylation (hypomethylation) in gene promoter regions can lead to increased gene expression [33]. During an acute inflammatory response, this mechanism is crucial for the rapid induction of genes involved in the immune response. For instance, hypomethylation of the promoter regions of genes encoding key pro-inflammatory cytokines such as TNF-α, IL-1β, and IL-6 has been observed to drive their increased expression in neurodegenerative diseases, including amyotrophic lateral sclerosis (ALS), Alzheimer’s (AD), and Parkinson’s disease (PD). For instance, decreased methylation of the gene promoter region of APP and APOE occurs in the brain of individuals with AD, typically leading to the overexpression of inflammatory molecules, further causing amyloid deposits [34]. Similarly, a reduction in methylation levels also enables the activation of microglia and astrocytes, which potentially causes a vicious cycle of multiple disease pathologies and progression [35]. In addition, aging has been considered a major risk factor for AD development, and DNA methylation is typically altered with an increase in age. Additionally, it was also found that the DNA methylation level at the CpG site in the genome of human donors is positively correlated with chronological age, indicating that age-related DNA methylation alterations highly affect gene expression in the brain [36].

In other neurodegenerative diseases like PD and ALS, acute inflammatory responses are similarly linked to DNA hypomethylation, driving the upregulation of pro-inflammatory interleukins, other cytokines, and toxic mediators of neurodegeneration. Studies have shown that reduced levels of methylation in the promoter region of TNF-α in the substantia nigra pars compacta can increase the susceptibility of dopaminergic neurons to TNF-α-mediated inflammatory reactions [18,37]. Although direct evidence for hypomethylation of the promoters of IL-1β and Il-6 in PD is not as extensively detailed as for TNF-α, the general principle that hypomethylation drives increased expression of these cytokines in inflammatory contexts strongly suggests a similar mechanism in PD.

While specific instances of hypomethylation of IL-1β, IL-6, or TNF-α promoters in ALS are still an active area of research, evidence points to broader epigenetic modification through differential methylation of immune-related regions in ALS individuals, which correlates with the spontaneous production of these cytokines. The presence of aberrant DNA and RNA methylation in the spinal cord and skeletal muscle of ALS models and affected individuals further supports the role of epigenetic dysregulation in the inflammatory processes linked to ALS [38]. This suggests that hypomethylation, as a general mechanism for gene activation, likely contributes to the upregulation of these pro-inflammatory mediators in ALS. In line with this, the findings presented here reveal increased expression of DNA methyltransferases (*Dnmt1*, *Dnmt3a*, and *Dnmt3b*), which initially contrasts with the observed hypomethylation in the promoter regions of BH4 pathway-related genes. This apparent contradiction suggests that additional regulatory mechanisms may be influencing DNA methylation dynamics. One possibility is competition with active demethylation enzymes, such as TET2, which can mediate local demethylation even in the presence of globally elevated DNMT levels. Notably, we observed similar region-specific epigenetic patterns in our previous work on the impact of neuroinflammation on Sonic Hedgehog signaling, where distinct brain regions exhibited divergent expression profiles of TET enzymes, suggesting that local microenvironmental cues may dictate the balance between methylation and demethylation pathways [37]. Additionally, specific CpG sites within the BH4 gene promoters may be selectively protected or demethylated due to chromatin accessibility or transcription factor occupancy, resulting in a locus-specific rather than global methylation pattern. It is also plausible that the hypomethylation observed is not a primary molecular trigger but rather a downstream consequence of neuroinflammatory processes, as inflammatory cytokines are known to reshape the epigenetic landscape and modulate DNA methylation states. These alternatives underscore the complexity of epigenetic regulation in the context of inflammation and highlight the need for further mechanistic studies to elucidate the causal relationships underlying BH4 gene expression changes.

While inflammation can initially be a protective response to injury or damage, its chronic and excessive activation contributes to neuronal dysfunction and cell death [for a review see [39]]. Indeed, the hippocampus is recognized as a region of the brain with heightened vulnerability to sustained immune system activation and neuroinflammatory processes. This susceptibility is attributed to several unique features of the hippocampus, including its high degree of plasticity, ongoing neurogenesis (particularly in the dentate gyrus), and extensive interconnections with immune signaling pathways. The hippocampus is a primary site for neuroimmune interactions, with cytokines and chemokines playing critical roles in modulating both normal hippocampal function and its response to injury or inflammation. These same mechanisms that underlie its plasticity also contribute to its vulnerability to immune-mediated insults, leading to dysregulation of neurogenesis, synaptic function, and cognitive processes such as learning and memory during sustained immune activation [40].

Here, it was shown that 24 h after an acute challenge with LPS, neuroinflammation, oxidative stress, and sustained activation of the BH4 biosynthetic pathway were induced. TNF-α, IL-1β, and hydrogen peroxide are three of the main stimulators of BH4 synthesis, positively regulating the expression of *Gch1* and favoring the development of inflammation [41,42]. Increased levels of BH4 can, in turn, further promote the development and persistence of inflammation. BH4 is a critical cofactor for nitric oxide synthases, including inducible NOS (NOSII), which produces high levels of NO to support Th1-mediated inflammatory responses [41,43]. Although NO has diverse physiological roles, under inflammatory conditions, excessive NO production contributes to oxidative stress and tissue damage, thereby intensifying the inflammatory response. This establishes a vicious cycle: inflammation induces the release of TNF-α and IL-1β, which stimulate BH4 synthesis through the upregulation of *Gch1*, and the resulting increase in BH4 further amplifies the inflammatory process. This mechanism highlights the complex interplay between inflammatory mediators and metabolic pathways in the central nervous system. Notably, it has been shown that BH4 is essential for immune cell proliferation and tissue infiltration, as it supports mitochondrial function [11]. Moreover, elevated BH4 levels have been reported in multiple preclinical models characterized by chronic inflammation [11,44,45]. Our group and others have shown that the LPS-induced acute inflammatory response is generally rapid, with cytokine levels often peaking within a few hours (e.g., 1.5 to 4 h) and then declining [46,47]. While cytokine levels may return to baseline within 24–48 h for some, other inflammatory markers or cellular changes can persist longer [48]. In this study, we demonstrated that several markers of inflammasome activation, oxidative stress, and BH4 biosynthesis were upregulated 24 h after the LPS challenge, and these sustained alterations may be driven, at least in part, by DNA hypomethylation.

Acute LPS stimulation has been shown to upregulate the expression of all *Dnmt* isoforms and *Tet2* in the hippocampus, leading to differential regulation of genes involved in the Hedgehog signaling pathway, which modulates cell proliferation and differentiation during inflammation [37]. In line with these findings, we demonstrated that a single LPS exposure event induced changes in the expression of key enzymes responsible for DNA methylation in the hippocampus. Specifically, we observed increased expression of *Dnmt1*, *Dnmt3a*, *Dnmt3b*, and *Tet2*, accompanied by decreased expression of *Tet1* and *Tet3*. These transcriptional changes support our hypothesis that DNA hypomethylation of the *Gch1* and *Ptps* promoter regions following acute inflammation may underlie the sustained upregulation of BH4 biosynthesis. Elevated BH4 levels, in turn, may enhance immune cell activity and contribute to the maintenance of an inflammatory state. For instance, our group previously reported that increased intracellular BH4 levels promote heightened immune responsiveness and cellular infiltration [11]. Additionally, hypomethylation of the *Gch1* gene has also been demonstrated in the spinal cord of animals subjected to chronic restraint stress, an experimental model that also leads to an inflammatory response in the brain, leading to an increase in gene expression and protein levels of BH4 biosynthetic enzymes [49]. Furthermore, regulation of the BH4 pathway through DNA hypomethylation may have significant implications for neurotransmitter metabolism besides the immune response. BH4 is also a critical cofactor for the synthesis of the monoamine neurotransmitters dopamine and serotonin, which play essential roles in mood regulation, cognition, and overall brain function [50]. In this context, evidence indicates that cerebrospinal fluid levels of BH4 are inversely associated with the progression of neurodegeneration in patients with PD [51]. This observation suggests that DNA hypomethylation in the promoter regions of genes involved in BH4 synthesis may represent a compensatory mechanism aimed at increasing BH4 availability, potentially to support the production of neurotransmitters such as dopamine and serotonin. However, as inflammation progresses, BH4 may become increasingly diverted to other biological pathways, notably NO synthesis, thereby contributing to oxidative stress and sustaining the inflammatory response.

It is known that dopaminergic neurons are prone to oxidative stress, mitochondrial dysfunction, and inflammation, particularly in the context of BH4 metabolism, arising from a convergence of biochemical, cellular, and metabolic factors that are less pronounced in serotonergic neurons. BH4 is synthesized at high levels in dopaminergic neurons (for a review see [1]). Under inflammatory conditions, BH4 levels can be further upregulated, especially in the presence of activated microglia, leading to increased dopamine synthesis and turnover. However, BH4 itself is redox-active and prone to autoxidation, generating reactive oxygen species (ROS) and contributing to oxidative stress. This is particularly problematic in dopaminergic neurons, where the combination of high cytosolic dopamine and elevated BH4 creates a microenvironment susceptible to the formation of toxic dopamine quinones and other oxidative products [52]. Experimental evidence demonstrates that BH4-induced cytotoxicity is dopamine-dependent: increasing cellular dopamine content heightens vulnerability, and non-dopaminergic cells only become susceptible to BH4 toxicity when exposed to dopamine [52,53]. Further compounding this vulnerability, BH4 exposure selectively induces cyclooxygenase-2 (COX-2) expression in dopaminergic cells, which in turn promotes dopamine oxidation and amplifies oxidative stress. Inhibition of COX-2 attenuates these effects, underscoring the role of inflammation-driven enzymatic pathways in dopaminergic neurodegeneration [54]. Dopaminergic neurons of the substantia nigra pars compacta are also distinguished by their exceptionally high metabolic rates and complex axonal arborization, which demand elevated mitochondrial oxidative phosphorylation and ATP production. This high bioenergetic demand results in a greater basal level of mitochondrial ROS and a reduced reserve capacity to buffer additional metabolic or oxidative insults. These neurons are therefore more susceptible to mitochondrial dysfunction, as seen in systems to model Parkinson’s disease, where impairments in ATP production or increased oxidative stress preferentially damage these cells [55,56]. In contrast, serotonergic neurons appear to be relatively spared under similar conditions. Non-dopaminergic neurons do not accumulate dopamine or BH4 to the same extent and are less affected by these specific oxidative and metabolic stressors [52,53,54,55,56].

## 5. Conclusions, Limitations, and Further Directions

Together, our results provide evidence that acute neuroinflammation induced by LPS promotes a coordinated neuroimmune and oxidative response, disrupts dopaminergic homeostasis, and upregulates BH4 biosynthesis in the hippocampus. Importantly, we demonstrate for the first time that this metabolic adaptation is associated with epigenetic remodeling, including promoter hypomethylation of BH4-related genes and altered expression of key DNA methylation and demethylation enzymes. These findings are consistent with recent studies emphasizing the role of DNA methylation in regulating transcriptional responses to inflammatory and oxidative stress in the brain [57,58]. The epigenetic regulation of BH4 metabolism may represent a novel mechanism by which the brain adapts its redox and neurotransmitter balance during immune activation. Given the essential roles of BH4 in neurodevelopment, immune modulation, and neurotransmission, our findings may have broader implications for understanding the pathophysiology of neuropsychiatric and neurodegenerative disorders characterized by chronic inflammation and epigenetic dysregulation. Future studies should investigate the persistence of these epigenetic marks beyond the acute phase, their cell type specificity, and their potential reversibility. Additionally, targeting BH4 metabolism or its epigenetic regulation could represent a promising therapeutic strategy for modulating neuroinflammation and associated behavioral impairments. Finally, we acknowledge that the use of only male rodents in this study limits the generalizability of the findings, as sex-specific epigenetic responses to inflammation cannot be excluded. Future investigations will be necessary to determine whether the observed epigenetic modifications are sex-dependent.

## Figures and Tables

**Table 1:** qPCR primer sequences and amplification conditions.

Gene (ID)	Primer	5′-3′ Sequence	Reaction Conditions	Product Size (bp)
***Nrf2 (18024)***	Forward	ATG GAG CAA GTT TGG CAG GA	95 °C-15 s; 61 °C-30 s; 72 °C-30 s	456
	Reverse	GCT GGG AAC AGC GGT AGT AT		
***Nlrp3 (216799)***	Forward	ATT ACC CGC CCG AGA AAG G	95 °C-15 s; 58 °C-30 s; 72 °C-30 s	141
	Reverse	TCG CAG CAA AGA TCC ACA CAG		
***Asc1 (104385)***	Forward	AGA CAT GGG CTT ACA GGA	95 °C-15 s; 60 °C-30 s; 72 °C-30 s	256
	Reverse	CTC CCT CAT CTT GTC TTG G		
***Caspase 1 (12362)***	Forward	TGA AAG AGG TGA AAG AAT T	95 °C-15 s; 59 °C-30 s; 72 °C-30 s	386
	Reverse	TCT CCA AGA CAC ATT ATC T		
***Il1b (16176)***	Forward	GAC CTT GGA TGA GGA CA	95 °C-15 s; 60 °C-30 s; 72 °C-30 s	183
	Reverse	AGC TCA TAT GGG TCC GAC AG		
***Il10 (16153)***	Forward	CCA AGC CCT TAT CGG AAA TGA	95 °C-15 s; 60 °C-30 s; 72 °C-30 s	163
	Reverse	TTT TCA CAG GGG AGA AAT CG		
***Il18 (16173)***	Forward	ACT TTG GCC GAC TTC ACT GT	95 °C-15 s; 60 °C-30 s; 72 °C-30 s	125
	Reverse	GGG TTC ACT GGC ACT TTG AT		
***Hmox1 (15368)***	Forward	CACGCATATACCCGCTACCT	95 °C-15 s; 61 °C-30 s; 72 °C-30 s	175
	Reverse	CCAGAGTGTTCATTCGAGCA		
***Ikbkb (16150)***	Forward	TCACTTGTCTCCTGGTGCTG	95 °C-15 s; 61 °C-30 s; 72 °C-30 s	234
	Reverse	TGTGCAGCAGATTTCCTTTG		
***Dnmt1 (13433)***	Forward	CCT TTG TGG GAA CCT GGA A	95 °C-15 s; 63 °C-30 s; 72 °C-30 s	240
	Reverse	CTG TCG TCT GCG GTG ATT		
***Dnmt3A (13435)***	Forward	GAG GGA ACT GAG ACC CCA C	95 °C-15 s; 63 °C-30 s; 72 °C-30 s	216
	Reverse	CTG GAA GGT GAG TCT TGG CA		
***Dnmt3B (13436)***	Forward	AGC GGG TAT GAG GAG TGC AT	95 °C-15 s; 63 °C-30 s; 72 °C-30 s	91
	Reverse	GGG AGC ATC CTT CGT GTC TG		
***Tet1 (52463)***	Forward	GAG CCT GTT CCT CGA TGT GG	95 °C-15 s; 65 °C-30 s; 72 °C-30 s	367
	Reverse	CAA ACC CAC CTG AGG CTG TT		
***Tet2 (214133)***	Forward	AAC CTG GCT ACT GTC ATT GCT CCA	95 °C-15 s; 65 °C-30 s; 72 °C-30 s	211
	Reverse	ATG TTC TGC TGG TCT CTG TGG GAA		
***Tet3 (194388)***	Forward	GTC TCC CCA AGT CCT ACC TCC G	95 °C-15 s; 63 °C-30 s; 72 °C-30 s	137
	Reverse	GTC AGT GCC CCA CGC TTC A		
***Gch1 (14528)***	Forward	TGT TGG TGT GTG CCT TGT CT	95 °C-15 s; 60 °C-30 s; 72 °C-30 s	191
	Reverse	TGT CAG CCA GAG GGA GAA CT		
***Ptps (19286)***	Forward	CTG CAT GTG GTT TTG GTG AG	95 °C-15 s; 60 °C-30 s; 72 °C-30 s	178
	Reverse	TTA ACG GCT GAG CCA TCT CT		
***Spr (20751)***	Forward	TAG AGG TGG GAA GAG CAG GA	95 °C-15 s; 59 °C-30 s; 72 °C-30 s	190
	Reverse	CCA GTG AAC ACC ACA GGA TG		
***Dhfr (13361)***	Forward	GAG GTC CAG GAG GAA AAA GG	95 °C-15 s; 60 °C-30 s; 72 °C-30 s	209
	Reverse	ATC CCC AGG ATC ACA AAA CA		
***b-Actin (11461)***	Forward	TCT TGG GTA TGG AAT CCT GTG	95 °C-15 s; 58 °C-30 s; 72 °C-30 s	82
	Reverse	AGG TCT TTA CGG ATG TCA ACG		
***Gapdh (14433)***	Forward	AGG CCG GTG CTG AGT ATG TC	95 °C-15 s; 58 °C-30 s; 72 °C-30 s	530
	Reverse	TGC CTG CTT CAC CAC CTT CT		

## Data Availability

The raw data supporting the conclusions of this article will be made available by the authors on request.

## Supplementary Materials

The following supporting information can be downloaded at https://www.mdpi.com/article/10.3390/brainsci15080880/s1, Table S1. Number of animals utilized for each measurement.
