# Supplementary material for: Resolution of Lipopolysaccharide-Induced Inflammation Followed by DNA Hypomethylation and Increased Tetrahydrobiopterin Biosynthesis in Mouse Hippocampus
Source: Brain Sci. 2025 Aug 18;15(8):880. doi: 10.3390/brainsci15080880 (PMC12385037; doi:10.3390/brainsci15080880)
Supplement: Supplementary file 1 [file brainsci-15-00880-s001.zip › brainsci-3756599-supplementary.pdf]

Supplementary material

Table S1. Number of animals utilized for each measurement

Measured parameter	Sample size (n)
Locomotor activity (Open field test)	6
Neopterin (Plasma)	8-9
Neopterin (Hippocampus)	5
Dopamine and serotonin levels	5
Nitrites, ROS and TBA-RS levels	5
Gene expression	5
DNA methylation	5

ROS: Reactive oxygen species; TBA-RS: Thiobarbituric acid reactive substances
